# A Review of Fluid Bolus in Critically Ill Patients After Initial Volume Expansion: Bayesian Probability Analysis and Case Studies

**DOI:** 10.7759/cureus.59517

**Published:** 2024-05-02

**Authors:** Sharad Patel, Nitin Puri, Shawana Hussain, Jean-Sebastien Rachoin, Adam Green

**Affiliations:** 1 Critical Care, Cooper University Hospital, Camden, USA

**Keywords:** monte-carlo simulation, refractory hypotension, sepsis treatment, fluid bolus, bayes theorem, shock

## Abstract

Introduction

Fluid resuscitation is a crucial intervention for the management of critically ill patients. However, after initial volume expansion, the advantages of fluid bolus administration remain controversial. Our aim was to investigate the probabilistic reasoning against fluid bolus administration in critically ill patients after initial volume expansion. We then applied this reasoning to two hypothetical case studies that evaluated the benefits and risks associated with a fluid bolus for each patient.

Methods

We analyzed data from 12 previously published studies, totaling 334 patients, on fluid responsiveness in critically ill patients. Owing to differences in these studies, we used a Monte Carlo simulation based on their parameters to improve our Bayesian prior, generate strong estimates, and address uncertainty. Using the established Bayesian prior for volume responsiveness, we scrutinized two hypothetical case studies employing Bayesian mathematical notation to assess the pre-test probability, posterior probability, and likelihood ratios in patients with septic shock.

Results

The Monte Carlo simulation yielded a mean response rate of 0.54 (SD = 0.026), suggesting that only approximately 54% of patients were responsive to fluid bolus administration. These results had an effective sample size of 17,204 and an R-hat value of 1, demonstrating the reliability of our results. In our Bayesian case studies, we demonstrate the low probabilities of volume and VO_2_ responsiveness over time using common bedside testing.

Conclusion

Our analysis shows that the pretest and posttest probabilities for volume responsiveness following initial fluid resuscitation are low. Additional bedside testing should be pursued before administering additional volume. This approach emphasizes the importance of evidence-based decision-making in the management of critically ill patients to optimize patient outcomes and minimize potential risks.

## Introduction

Fluid bolus administration is a common intervention in the intensive care unit and the efficacy of post-initial volume expansion remains controversial [1,2]. A commonly cited review by Michard and Teboul utilized 12 studies and found that only 52% of the patients were identified as fluid responders as defined by an increase in stroke volume and cardiac output [3]. However, a more physiologically insightful approach involves assessing DO_2_ (oxygen delivery) and VO_2_ (oxygen consumption). These markers provide a deeper understanding of the balance between oxygen supply and demand in critically ill patients but are more resource-laden and therefore not frequently utilized. A few studies have studied VO_2_ responsiveness using the P(v-a)CO_2_/CaO_2_ ratio after fluid loading in septic shock patients [4,5]. About 50% of patients with septic shock are volume responders and a little more than half of those patients increase their VO_2_ by more than 15% after fluid administration (roughly 30% of total patients) [5,6]. This suggests that fluid administration in septic shock requires a probabilistic framework to identify the patients that would benefit.

Part of the problem clinicians face is it is unclear when fluid should be administered post-initial resuscitation. In septic shock, initial fluid expansion is protocolized but the decision to provide additional fluid is a complicated one. A patient should receive fluid ideally when all of the following criteria are met: the patient shows signs of intravascular depletion (hypotension), volume responsiveness (cardiac output increase of 10-15%) [3,7,8], better tissue perfusion (DO2 increase), and improved tissue oxygenation (VO_2_ increase). In addition, fluid administration should have a lasting effect. Unfortunately as shown by the Andromeda Shock trial, after each hour of hospitalization, the probability of volume responsiveness decreases [9].

The potential for fluid responsiveness needs to be balanced with the chance of negative side effects due to fluid administration. A positive fluid balance has been correlated with increased mortality in sepsis [10-12] and has hinted at increased mortality in other types of shock [13-15], while patients treated with a conservative fluid strategy are less prone to developing worsening renal failure and have better outcomes [16,17]. However, human decision-making often fails to integrate probabilistic reasoning, relying on intuitive heuristics instead [18,19].

Thus, a structured approach to assessing ongoing fluid utility is imperative. We propose an explicit Bayesian framework integrating conditional probabilities to optimize fluid administration. The steps include the following: evaluate for sustained hypotension after initial resuscitation; estimate the prior probability of volume responsiveness from existing evidence; incorporate time-dependent and diagnostic test factors to update probability; compare the probability of benefit against risks like worsening acute kidney injury (AKI); and, where suggested, stop fluids and utilize alternatives.

Clinicians often persist with ingrained interventions like fluid boluses, anchoring on habitual norms rather than integrating new evidence to update probabilities. Thus, despite declining utility over time, fluids remain a knee-jerk resuscitation reflex. Our study aimed to provide an explicit Bayesian framework to inform decision-making around fluid bolus administration.

## Materials and methods

Overview

This review will utilize a Monte Carlo simulation to determine the percentage of patients likely to be fluid responders. Monte Carlo simulation is a mathematical technique that allows a small sample size to provide robust results typical of much larger studies. This allows us to use a commonly cited and well-known study by Michard and Teboul to estimate the percentage of patients likely to be fluid responders [3]. Once this is determined, a Bayesian analysis will be applied to two fictitious cases to demonstrate the clinical utility of these results.

To address the variability and diversity in the data from the 12 studies on volume responsiveness [3], we utilized a statistical technique known as the Monte Carlo simulation. This approach allowed us to obtain a more robust prior for our subsequent Bayesian case analysis. The 12 studies we reviewed provided valuable insights into volume responsiveness; however, they also presented a range of probabilities due to variations in patient populations, fluid types, and measurement methods. By simulating numerous trials, each representing a potential response to fluid bolus administration in critically ill patients, we constructed a distribution of probabilities for volume responsiveness. To conduct the Monte Carlo simulation, we leveraged PyMC4, a Python library specifically designed for probabilistic programming. This software ensured the consistency and reproducibility of the simulation. In the simulation, each trial was treated as a random variable to capture the observed variability from the original studies. By generating a multitude of trials, we encompassed a wide range of volume-responsiveness probabilities observed in the studies. This comprehensive approach provided us with a deeper understanding of the potential outcomes of fluid bolus administration in critically ill patients. By employing Monte Carlo simulation, we aimed to establish a more robust prior for our subsequent Bayesian case analysis. This prior study serves as a foundation for further investigations, enabling us to assess the potential benefits and risks of fluid bolus administration in a more nuanced and individualized manner.

Bayesian case analysis

As physicians, making informed decisions under uncertainty is part of our daily practice, guiding volume administration for a patient with septic shock requires precisions, which Bayes’ theorem can provide. Bayes' theorem offers a powerful framework for incorporating new evidence into our existing knowledge, enhancing our diagnostic precision. At its core, Bayes' theorem calculates the probability of a condition or hypothesis (H) given new evidence (E). It starts with what we already suspect (prior probability) and updates it based on how likely we are to see the evidence if the condition were true. The formula simplifies to: P(H|E) = (P(E|H) * P(H)) / P(E). Definitions of these terms are as follows. Prior probability (P(H)): our initial belief about the presence of a disease before considering new test results; (P(E)): The probability that new evidence occurs; likelihood (P(E|H)): the probability of observing the current evidence if the hypothesis is true; posterior probability (P(H|E)): our updated belief about the disease's presence after incorporating the test result.

Understanding odds is crucial in the Bayesian framework. Odds provide a more intuitive way to think about probabilities, calculated as odds = probability / (1 - probability). This conversion is key for applying Bayes' theorem in clinical settings, making it easier to update our beliefs with new data.

Consider diagnosing myocarditis in a patient presenting with chest pain and fatigue. Based on your clinical experience and regional prevalence, you estimate a 30% prior probability of myocarditis in such cases. Pretest probability: 30% or 0.30; pretest odds: 0.30 / 0.70 = 0.43. You order a cardiac MRI, which is 90% accurate for diagnosing myocarditis when positive. The likelihood ratio (LR) for a positive result might be around 9, considering the test's specificity and sensitivity. After receiving a positive MRI result, you update your diagnosis: post-test odds: 0.43 * 9 = 3.87; post-test probability: 3.87 / (1 + 3.87) = 0.79 or 79%. This example illustrates how Bayes' theorem helps us refine the probability of myocarditis from an initial 30% to 79% after incorporating the MRI results, significantly affecting our clinical decisions.

By mastering the conversion from probabilities to odds and applying Bayes' theorem, physicians can enhance their diagnostic accuracy, embracing a more nuanced understanding of patient care in the face of uncertainty. This approach doesn't require deep statistical knowledge but an appreciation for how evidence shapes our clinical judgments.

To perform a strong Bayesian analysis, you need a robust prior probability. To ensure our Bayesian analysis stands on solid ground, we employ a robust prior probability, derived from Monte Carlo simulations. We selected two simple fictitious case studies that illustrated the application of Bayesian analysis for patients with septic shock. We evaluated the posterior probabilities of volume and VO_2_ responsiveness. For volume responsiveness, we used 54% derived from our Monte Carlo analysis (see results below). In the scenario where volume responsiveness was measured at a delay from presentation, we used numbers provided by the Andromeda Shock trial [9]. The values are listed in Table 1. For VO_2_ responsiveness, we combined the results of two studies to use a value of 28.9% (Table 2) [3,6]. Other values required to complete the calculations in our case studies include the LR- of a passive leg (0.09) [20], LR- for volume responsiveness with a distended IVC with minimal variation (0.44) [20], and the prior probability of AKI in septic shock (0.64) [21].

**Table 1:** Probability of volume responsiveness over time in Andromeda shock trial

Time (hours)	Probability of volume responsiveness
0	57%
2	22%
4	11%
6	10%
8	3%

**Table 2:** Calculation of VO2 responders

Study	Total patients	Volume responders	VO_2_ responders
Study 1 (Mallat et al.)^[6]^	98	52%	30%
Study 2 (Monnet et al.)^[^^5]^	51	49%	27%
Combined data	149	N/A	29%

## Results

Monte Carlo simulation

Our Monte Carlo simulation indicates that approximately 54% of patients were responsive to fluid bolus administration. To understand the range of possible response rates, we calculated the 95% highest density interval (HDI) ranging from 0.491 to 0.589. This interval represents the range within which we can be 95% confident that the true response rate is within. This accounts for the inherent uncertainty and variability in the response rate, highlighting the need for individualized decision-making when considering fluid bolus administration. These results are presented in Table 3.

**Table 3:** Monte Carlo simulation results for volume responsiveness

Metric	Value	Description
Mean response rate	0.54 (SD = 0.026)	The mean percentage of patients responsive to fluid bolus administration.
95% highest density interval (HDI)	0.491-0.589	The interval containing 95% of the posterior probability density indicates the response rate range.
Effective sample size (ESS) bulk	17204	The number of independent samples in the simulation for bulk, with higher values indicates a more reliable estimation.
Effective sample size (ESS) tail	28544	The number of independent samples in the simulation for the tail, with higher values, indicates a more reliable estimation.
R-hat	1.0	The potential scale reduction factor assesses the simulation's convergence, with values close to one signifying convergence.

To assess the reliability of our simulation results, we considered two measures: effective sample size (ESS) and R-hat values. The ESS was assessed in the bulk and tail of the distribution, representing the central portion and outer edges, respectively. With values of 17,204 for the bulk and 28,544 for the tail, these figures reflect the number of independent samples in both regions, indicating reliable estimates and robustness across the entire distribution. In addition, the R-hat value, which measures the convergence of the simulation, was approximately 1. This suggests that the simulation results are reliable. In summary, the Monte Carlo simulation allowed us to obtain more reliable estimates of volume responsiveness by considering the variability and diversity of the data from the 12 studies. Our results showed that, on average, approximately 54% of patients were responsive to fluid bolus administration. The 95% highest density interval provided a range within which we could be 95% confident that the true response rate lies. The highly effective sample size and close-to-1 R-hat values indicate the reliability and convergence of our simulation. These findings contribute to a better understanding of individualized decision-making regarding fluid bolus administration in critically ill patients.

Case analysis

Case Study 1: Bayesian Analysis in a Septic Shock Patient

A 55-year-old patient with a history of diabetes presents with hypotension and signs of a urinary tract infection. Despite receiving a 30 cc/kg fluid bolus, the patient remains hypotensive and is quickly assessed by the ICU team. The team performs a passive leg raise, which is negative, suggesting low-volume responsiveness. Utilizing equations from the methods section, the analysis yields a posterior probability for volume responsiveness at 9.55%, indicating a low likelihood of positive response to further fluid administration. This low probability is primarily due to the negative passive leg raise test.

If evaluation occurred two hours post-hospitalization, reflecting on data from the Andromeda Shock trial [9], the prior probability of volume responsiveness adjusts to 22%. This change significantly impacts the posterior probabilities, reducing the volume responsiveness to 2.48% and VO_2_ responsiveness to 0.70%. These results are presented in Table 4.

**Table 4:** Probability of volume responsiveness of case study 1

Physiological response	Prior probability	LR-	Posterior odds	Posterior probability
Volume responsiveness (1 hr)	54%	0.09	0.1056	9.55%
Volume responsiveness (2 hr)	22%	0.09	0.0254	2.48%
VO_2_ responsiveness (1 hr)	29%	0.09	0.0368	3.55%
VO_2 _responsiveness (2 hr)	14.5%	0.09	0.0185	1.82%

This analysis reinforces the utility of a negative passive leg raise test in predicting fluid responsiveness, highlighting the critical role of timely assessment in clinical decision-making.

Case Study 2: Bayesian Analysis in an Early-Evaluated Septic Shock Patient

Early evaluation of a septic shock patient reveals an inferior vena cava (IVC) distended by 2.3 cm with minimal variation, indicating potential fluid overload. Applying Bayesian analysis to these clinical findings, we calculate posterior probabilities for volume and oxygen consumption responsiveness. Given the challenges in directly calculating AKI risk from IVC observations, we reference a known prior probability of AKI in septic shock patients at 64% [21]. The calculated probability of volume responsiveness is 30.9%, with an oxygen consumption responsiveness of 13.5%, as detailed in Table 5.

**Table 5:** Probability of volume responsiveness of case study 2 AKI: acute kidney injury

Condition	Prior probability	LR+	LR-	Posterior probability
Volume responsiveness	54%	0.44	2.28	29.9%
Oxygen consumption responsiveness	25%	0.44	1.81	12.9%
AKI	64%			

This case illustrates that despite a higher prior probability of AKI (64%), the observed IVC distension and minimal variation suggest a cautious approach to fluid bolus administration due to the potential risk of worsening AKI. Alternative management strategies, focusing on personalized hemodynamic targets or advanced monitoring techniques, may better serve patient outcomes in such scenarios.

## Discussion

Our analytic approach provides a roadmap for integrating probabilistic reasoning at the bedside despite clinical uncertainty. Leveraging easily obtained pre-test odds and likelihood ratios offers a template for evidence-based decision-making via concise Bayesian calculations. We demonstrated how conditional probabilities over time along with test characteristics can provide objective quantification of whether meaningful fluid responsiveness persists. Comparing volume responsiveness probabilities to expected harm risks like acute kidney injury facilitates weighing whether to prioritize alternative management strategies.

The overarching goal of the de novo Monte Carlo simulation was to establish a more robust Bayesian prior to study heterogeneity. However, clinicians need not employ complex methods themselves. This analysis underscores how basic serial posterior updating can optimize practice despite engrained heuristics. Our case studies exemplify expected probability trajectories, intentionally selected to encourage deliberation amidst potential inertia. While decreased responsiveness over time and with positive/negative tests is well-established, seeing mathematical quantification provides a behavioral mirror to reconsider reflexive actions. By elucidating the risk/benefit balances mathematically through widely accessible means, this style of transparent analysis paves the way for improved mindfulness. As insights on goal-directed/precision care strategies grow, probabilistic reasoning offers a roadmap for reasoned adoption to improve patient outcomes.

One potential criticism is our reliance on a single review (Michard and Teboul) to inform the Bayesian prior [3]. However, this study remains highly influential and relevant, as evidenced by a 2016 clinical review citing it as the "most frequently cited systematic review" on fluid responsiveness [1]. Our intention was to establish a reasonable prior, not an exhaustive review. Though not the most recent, the Michard and Teboul review seems well-established as an influential reference on this topic.

Ultimately, this analysis shows how modern medicine can outgrow outdated habits. By making evidence-based thinking simple at the bedside, we can finally focus care on risks over reflexes. Just as probability shaped visionary fields like business, calculated odds must now guide clinical decisions daily. Although one small step for sepsis, the approach shown here demonstrates how all of medicine can enter a new era - where every patient benefits from analyzing personalized risks over one-size-fits-all rituals.

## Conclusions

This study shows how basic probability analysis can reveal when common interventions, like ongoing fluid boluses, offer little benefit. By calculating probabilities and updating priors using Bayes’ theorem, we can determine which septic patients are most likely to respond at the bedside. This evidence-based approach prevents low-yield rituals and allows personalized treatment focused on those with the highest probability of improvement. As medicine embraces calculated tradeoffs over one-size-fits-all maxims, all patients may one day benefit from objective, compassionate care aligned closely with their individual risks and preferences with the goal of ultimately improving outcomes.
